# Positive attitudes and negative expectations in lonely individuals

**DOI:** 10.1038/s41598-020-75712-3

**Published:** 2020-10-29

**Authors:** Gabriele Bellucci

**Affiliations:** Department of Computational Neuroscience, Max Planck Institute for Biological Cybernetics, 72076 Tübingen, Germany

**Keywords:** Human behaviour, Psychology

## Abstract

Loneliness is a central predictor of depression and major factor of all-cause mortality. Loneliness is supposed to be a warning signal prompting individuals to seek out social connections. However, lonely individuals seem to be less likely to engage in prosocial activities and are overall more socially withdrawn. Hence, it is yet unclear whether and how loneliness affects an individual’s social motivations. Prosocial attitudes and expectations about social interactions of lonely individuals might shed light on whether lonely individuals are more prone to connect or withdraw from social activities. Here, results from a large dataset (~ 15,500 individuals) provide evidence for both. In particular, lonely individuals indicate stronger altruistic attitudes, suggesting a positive tendency to build and maintain social bonds. However, they also report more negative expectations about others, as they believe their social partners be less fair and trustworthy, suggesting less favorable evaluations of social interactions. By highlighting an important link between loneliness, prosocial attitudes and social expectations, this work stresses the role of loneliness in social motivations, points to potential consequences for social behaviors, and proposes a mechanism for the paradoxical effects of loneliness on an individual’s social attitudes and expectations, with important implications for future basic and clinical research, as well as education, economics and public policy.

## Introduction

Loneliness is a worldwide public health concern affecting millions of people across the globe^1,2^. Approximately 15–30% of people suffer from prolonged feelings of loneliness with severe implications for physical and mental health^3^. In the next decades, increasing feelings of loneliness will dramatically inflate the incidence of depression, heart diseases and mortality in the general population^4,5^. Causes are multifactorial and not limited to the aging population, as loneliness affects younger individuals as well^6,7^. Most likely, they relate to an individual’s interpersonal attitudes and how social interactions unfold in the modern urban lifestyle^8,9^. However, the relationships between loneliness and social behaviors have gone largely unexplored.

Loneliness (or perceived social isolation) is characterized by unsatisfactory social relationships^10^. Loneliness is different from objective social isolation or social exclusion, where people are objectively shunned by others and experience feelings of anger and revenge^11,12^. In fact, being objectively isolated does not necessarily imply that individuals feel actually lonely (i.e., being alone is different from being lonely). Feelings of loneliness emerge when an individual perceives a lack of companionship and meaningful social relations, and are supposed to work as warning signal to prompt individuals to seek out social connections^13^. Hence, loneliness should motivate to prosocial behaviors that help build meaningful social ties and fulfill one’s need to belong^14–16^. In line with this, previous work has shown that individuals who experience momentary feelings of loneliness crave for social interaction^17^. However, there is also evidence that lonely individuals are more negatively biased toward others and more socially withdrawn^18,19^. Lonely individuals tend to be self-absorbed and their social skills are perceived as being of poorer quality^20,21^. Further, loneliness has been associated with social anxiety symptoms across childhood and adolescence, and experimentally-induced social isolation has been observed to decrease different prosocial behaviors such as donating, helping and cooperation^22^.

Previous studies have mainly focused on the negative effects of loneliness on health, unearthing the relationships between loneliness and different physical and mental disorders^23,24^. For instance, feelings of loneliness are a specific risk factor for depression and a genetic predisposition toward loneliness is associated with cardiovascular, psychiatric, and metabolic disorders^25,26^. However, these studies have left unexplored the associations between loneliness, prosocial attitudes and individual expectations about social partners. A better understanding of the relationships between loneliness and social motivations and expectations might provide valuable insights into the pathogenesis of chronic loneliness and how it increases the risk of several disorders. For instance, previous studies have shown a close link between individual expectations and mental health, as expectations of social rejection have been associated with depression, anxiety, and borderline personality disorder^27,28^, and expectations of hopelessness and self suicidal behavior have been observed to predict actual future suicidal behavior^29,30^.

Here, I investigated social expectations and attitudes of lonely individuals to gain insights into their propensity to be socially engaged or socially withdrawn. Social motivations (e.g., altruistic motives) and beliefs about others’ social behaviors (e.g., expectations of fair and reciprocal behaviors from others) were used as proxies for an individual’s propensity to prosocial behaviors and expectations about social partners' behaviors, respectively^31–33^. Using data from approximately 15,500 individuals, I tested the relationships between loneliness, social attitudes (as proxy for motivation to connect), and beliefs about social partners (as proxy for expectations about social interactions). If loneliness gates people’s resources to build meaningful relationships, greater feelings of loneliness should relate to a stronger willingness to behave altruistically and support others (e.g., helping). On the contrary, if loneliness heightens negative expectations about social interactions, greater feelings of loneliness should relate to less favorable expectations about others (e.g., their fairness).

## Results

### Loneliness and prosociality

**Figure 1:**
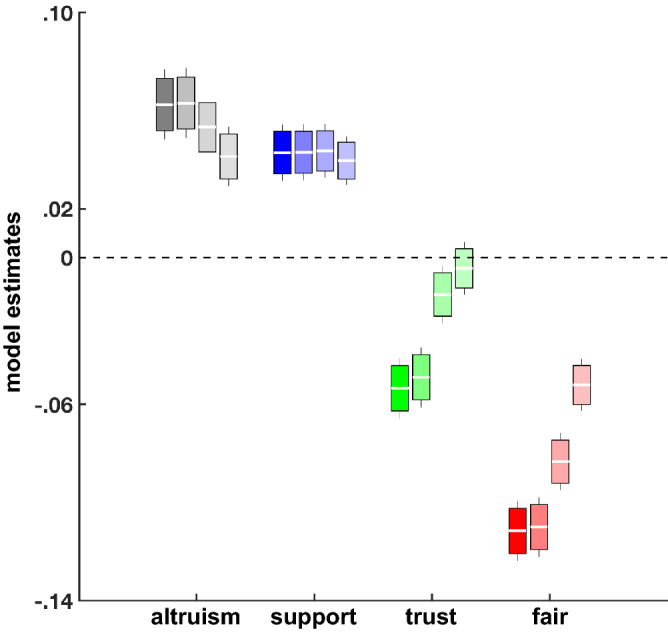
Regression model comparison. Differences in model estimates (with confidence intervals) for altruistic motives, social support, and beliefs about others’ trustworthiness and fairness in the four regression models (from left to right, model 1–4). Loneliness was positively associated with altruistic motives and social support, and negatively with trustworthiness and fairness expectations. Trustworthiness beliefs were not significant in the last model after introducing regressors for depression and helplessness (model 4).

First, I examined the relationships between loneliness and different prosocial preferences in regression model 1. Results show that lonelier individuals believe others to be less fair (*β* = − 0.11; standard error (SE) = 0.006; *p* < 0.0001) and trustworthy (*β* = − 0.05; SE = 0.006; *p* < 0.0001) and felt less compelled to comply with a reciprocity norm (*β* = − 0.02; SE = 0.007; *p* < 0.006; Fig. 1 and Table 1). However, lonelier individuals were also more willing to help others (*β* = 0.06; SE = 0.007; *p* < 0.0001) and support one’s social network (*β* = 0.04; SE = 0.006; *p* < 0.0001). These results are consistent with both the hypothesis that loneliness promotes negative expectations about others, loosening compliance with social norms, and with the hypothesis that it also increases one’s propensity to act prosocially and support others, making individuals more likely to have stronger prosocial attitudes.

**Table 1:** Regression analysis.

Predictors	Regression models			
	Model 1	Model 2	Model 3	Model 4
Intercept	− 0.16 (0.01)***	− 0.16 (0.01)***	− 0.06 (0.02)***	− 0.10 (0.01)***
**Social preferences**				
Altruism	0.06 (0.007)***	0.06 (0.007)***	0.06 (0.007)***	0.04 (0.006)***
Social support	0.04 (0.006)***	0.04 (0.006)***	0.04 (0.006)***	0.04 (0.005)***
Reciprocity	− 0.02 (0.007)**	− 0.02 (0.007)**	− 0.02 (0.006)*	− 0.01 (0.006)
Fairness	− 0.11 (0.006)***	− 0.11 (0.006)***	− 0.08 (0.006)***	− 0.05 (0.005)***
Trust	− 0.05 (0.006)***	− 0.05 (0.006)***	− 0.02 (0.006)*	− 0.004 (0.006)
**Social network quality**				
Overall social contact	–	− 0.09 (0.007)***	− 0.08 (0.006)***	− 0.06 (0.006)***
Meaningful social contact	–	− 0.04 (0.007)***	− 0.02 (0.007)**	− 0.02 (0.006)*
Contact seeking	–	0.001 (0.007)	0.01 (0.007)	0.01 (0.006)
**Health and life satisfaction**				
General health status	–	–	− 0.09 (0.007)***	− 0.02 (0.006)***
Life satisfaction	–	–	− 0.27 (0.009)***	− 0.10 (0.009)***
**Mental health**				
Depression	–	–	–	0.28 (0.008)***
Hopelessness	–	–	–	0.14 (0.007)***
**Biographical variables**				
Sex	0.02 (0.003)***	0.01 (0.003)***	0.01 (0.003)***	0.003 (0.003)
Age	− 0.0003 (0.0001)*	− 0.001 (0.0001)***	− 0.001 (0.0001)***	− 0.0004 (0.0001)***
Education	− 0.05 (0.007)***	− 0.04 (0.007)***	− 0.02 (0.006)**	− 0.01 (0.006)*
Employment status	− 0.002 (0.004)	0.01 (0.004)*	0.009 (0.003)*	0.003 (0.003)
Relationship status	0.05 (0.004)***	0.05 (0.004)***	0.04 (0.003)***	0.03 (0.003)***
Urban living	− 0.006 (0.006)	0.001 (0.006)	0.003 (0.005)	0.004 (0.005)

The predicted variable in all hierarchical regression models was feelings of loneliness (continuous dependent variable). The sex regressor was coded 1 = female and 0 = male; the employment status regressor was coded 1 = employed and 0 = unemployed; the relationship status regressor was coded 1 = single and 0 = in a relationship. Values represent $\beta$ values and standard errors in parentheses. *p < 0.05; **p < 0.01; ***p < 0.001.

Given the small effect sizes of the predictors (|*β*|< 0.12) and to test the importance of the relationship between prosociality and loneliness, the cross-validated performance of a model with only prosocial preferences (social model) was investigated with respect to how well such a model is able to predict individual levels of subjective feelings of loneliness. Implementing a bootstrap procedure, a regression model entailing only the regressors for social preferences was fitted to 20, 50 and 80% of the original dataset (training data). In every iteration, the fitted model was used to predict subjective values of loneliness of the left-out data (test data). Prediction error (i.e., standardized mean square error, smse) was computed as a measure of the model’s predictive performance. This procedure was repeated 10,000 times for each of the three different sample sizes (for a total of 30,000 predictions), yielding a distribution of the model’s mean cross-validated predictive performance. This distribution was compared to two predictive performance distributions: 1) one yielded by a null model, namely, the full model 1 trained on permuted labels (i.e., loneliness scores) instead of the true labels (permuted model); and 2) one yielded by a model containing only the biographical variables (biographical model), which represented a fair model for performance comparison given the similar effect sizes of the associations between biographical variables and feelings of loneliness (see Table 1).

**Figure 2:**
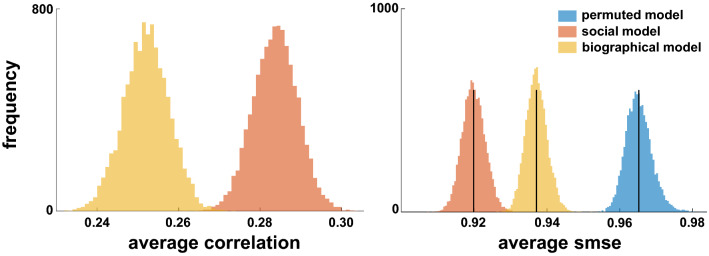
Cross-validated predictive performance. Average cross-validated predictive performance of the model with the regressors of interest (social model, orange) and with biographical regressors (biographical model, yellow) across the three training datasets with different sample sizes. Their predictive performance was compared to a null model with permuted labels (loneliness scores) as training data (permuted model, blue). Left are depicted average Pearson correlation coefficients between the model-based predictions and the true labels. Right are depicted the predictive performance distributions of the three models, that is, their standardized mean square errors (smse). Black lines represent mean predictive performance. Lower smse values represent better model's performance (smaller errors).

As can be seen in Fig. 2, the average Pearson correlation between the model-based predicted labels and the true labels was significantly higher for the social model (*r* = 0.28) as opposed to the biographical model (*r* = 0.25). Further, the average predictive performance (i.e., smse) of the social model (smse = 0.92) outperformed both the permuted (smse = 0.97) and biographical models (smse = 0.94). These results indicate a strong relationship between prosociality and loneliness that is able to predict subjective levels of loneliness in out-of-sample individuals.

### Loneliness and social contact

The stronger propensity to help and support others in lonelier individuals might be due to an overall reduced quality of their social contact, in particular, with relevant others. A second regression model adding variables related to an individual’s quality of social contact supports this hypothesis. In particular, lonelier individuals have overall less social contact (*β* = − 0.09; SE = 0.007; *p* < 0.0001), specifically with relevant others (*β* = − 0.04; SE = 0.007; *p* < 0.0001). However, in line with previous results, they were not less likely to seek social contact (*β* = 0.001; SE = 0.007; *p* = 0.910). Importantly, the regressors for altruistic motives (*β* = 0.06; SE = 0.007; *p* < 0.0001) and social support (*β* = 0.04; SE = 0.006; *p* < 0.0001) remained significant even after accounting for quality of social contact.

### Loneliness and health

As loneliness has been associated with poor health, it still is an open question whether more negative expectations in lonelier individuals are due to their generally worse health conditions, in particular their depression-like symptoms. Depressive feelings are here particularly relevant, because they are also characterized by more negative evaluations of social interactions and partners. Hence, one may argue that introducing measurements of health condition and depressive feelings could make the associations between loneliness and negative expectations disappear. The next results disconfirm this line of reasoning.

On the one hand, loneliness was indeed negatively associated with a worse health condition (*β* = − 0.09; SE = 0.007; *p* < 0.0001) and reduced life satisfaction (*β* = − 0.27; SE = 0.009; *p* < 0.0001). Moreover, loneliness was also associated with greater feelings of depression (*β* = 0.28; SE = 0.008; *p* < 0.0001) and helplessness (*β* = 0.14; SE = 0.007; *p* < 0.0001). On the other hand, however, all other relationships between loneliness and social preferences remained significant. The relationship between loneliness and trustworthiness expectations was the only relationship affected by the introduction of the depression and helplessness regressors in the last model (Fig. 1 and Table 1). These results suggest that the strong link between loneliness and prosocial preferences (both prosocial attitudes and social expectations) is not fully accounted for by depression-like feelings and general poor health conditions. Moreover, loneliness also reduces individual life satisfaction beyond the negative effects of depression, helplessness and poor health. This might confirm the specific link between feelings of loneliness and an individual’s negative expectations. In particular, life satisfaction is related to a person’s self evaluations and the introduction of the regressor for life satisfaction (together with health condition) in model 3 strongly reduced the effects of social expectations on loneliness (in particular, trustworthiness and fairness expectations) but not the effects of social motivations (i.e., altruism and social support), suggesting some common variance between social expectations and life satisfaction. Hence, this result indicates that loneliness is not only associated with more negative expectations about others but also about one’s self.

### Loneliness and other biographical variables

Biographical variables such as age, sex, education, urban living, employment and relationship status were controlled for in every regression model. Each of the these variables was significant in all models except for employment status and sex. In particular, women report greater feelings of loneliness but this relationship was no longer significant after accounting for depression-like feelings (*β* = 0.003; SE = 0.003; *p* = 0.278; Table 1). In summary, results from the last model indicate that younger adults (*β* = − 0.0004; SE < 0.0001; *p* = 0.011), singles (*β* = 0.03; SE = 0.003; *p* < 0.0001), and less educated individuals (*β* = − 0.01; SE = 0.006; *p* = 0.014) are more likely to suffer from greater feelings of loneliness.

## Discussion

Using a large dataset, this study investigates for the first time the relationships between loneliness, prosocial attitudes and individual expectations about social partners. Results show that loneliness is associated with a strong motivation to engage in prosocial behaviors, such as helping and supporting others, as well as less favorable expectations about social partners, such as their fairness and trustworthiness. Such relationships could significantly predict subjective levels of loneliness in out-of-sample individuals and remained significant after controlling for individual social network quality (e.g., frequency of contact with close others), and individual health conditions (e.g., depression and helplessness). These findings indicate that loneliness is characterized by both positive prosocial tendencies, likely reflecting a motivation to seek out social connections, and negative expectations about social interactions, likely reflecting a social evaluation bias that foster social withdrawal.

On the one hand, greater feelings of loneliness were associated with increased motivation to help and support others, and poorer quality of social contact. Importantly, even though lonely individuals tended to have less social contact with others, especially with close others, they were not found to be less likely to seek out social connections. These results well accord with the warning signal hypothesis suggesting that loneliness originates from unsatisfactory social relationships and prompts individuals to connect in order to satisfy their unfulfilled need to belong^34^. In particular, lonely individuals might engage in behaviors signaling positive social qualities (e.g., altruistic motives) that could help them increase the chances to establish meaningful and long-lasting social ties with others^35^.

On the other hand, loneliness was also related to more negative expectations about others’ trustworthiness and fairness, and to a reduced willingness to reciprocate. Such expectations might reflect more pronounced negative evaluations of social interactions in lonely individuals. These results chime with previous evidence showing that loneliness disrupts trust in others, cooperation and an individual’s sense of community^11,36,37^. In particular, a bias to negatively evaluate social interactions and social partners might induce negative attitudes (e.g., avoidance behavior) and negative affect (e.g., frustration and demotivation) that foster social withdrawal^3,38^. Alternatively, these negative expectations might indeed contribute to an individual’s willingness to engage in prosocial behaviors (e.g., I helped because I expected nobody else would) but might then corrupt the feedback evaluation that follows the social engagement (e.g., the behavior of the person I helped comes across as unthankful). Ultimately, these effects of loneliness on people’s social evaluations might impair their ability to successfully connect and form satisfactory, social bonds^39^.

The observed association between feelings of helplessness and greater feelings of loneliness might reflect such difficulties in successfully building a supportive social network. Having social ties of poor quality signals a threatening lack of social support that makes individuals feel more vulnerable and exposed to, and hence less able to cope with, life stressors. It has been shown that a supportive social network works as a social buffer with positive effects on an individual’s physical and mental well-being^40–43^. Hence, feelings of helplessness signal the absence of such soothing and supportive social fabric, and might as such become a source of stress and anxiety, heightening vigilance to threats in lonely individuals and so worsening their mental and physical health^38^.

Further, the negative association between loneliness and life satisfaction suggests that lonely individuals do not only evaluate more negatively their social partners, but also themselves. This is in line with previous studies showing that lonely individuals have lower self-regard, self-confidence and self-esteem^21,44,45^. Life satisfaction is low when the perceived discrepancy between the desired and the actual life circumstances is negatively loaded^46^. Importantly, evaluation processes are central to determining the negativity or positivity of such a discrepancy, as the mismatch between the desired and realized life outcomes relies on judgments about one’s life circumstances^47^. Harsher self-evaluations, which are, for instance, associated with less favorable judgments about what one has accomplished, might hence be heightened by stronger feelings of loneliness, contributing to an overall dissatisfaction with one’s own life.

Thus, the findings of the current work suggest that loneliness is associated with both increased altruistic motives and unfavorable expectations about social interactions, indicating that loneliness might be characterized by two mutually non-exclusive mechanisms. One the one hand, loneliness has a healthy effect on individuals by promoting the search for social contact and bonds (reflected by more positive social tendencies). On the other, it promotes a systematic evaluation bias that makes an individual consistently perceive her need to belong as left unsatisfied (due to more negative expectations about social interactions), thereby likely fostering social withdrawal and paving the way for depression-like feelings. Importantly, these two mechanisms do not have to work in parallel and might (and likely do) interact in quite complicated ways. For instance, lonely individuals might seek out social connections to satisfy their unmet social needs but their evaluation of the resulting social interactions might be so negatively biased that their attempts to connect end up confirming and strengthening their negative expectations. On the long run, this vicious circle might drift lonely individuals to increasingly withdraw themselves from the social sphere.

These mechanisms might explain preliminary evidence that lonely individuals tend to be more positive toward less close acquaintances but, paradoxically, more negative toward close others^21^. That is, lonely individuals might be motivated to meet new people but over time, they might form such negatively biased impressions of their acquaintances that they wish for their distance instead of their closeness. Furthermore, these mechanisms might be part of the psychological dynamics underlying the development of depression from chronic loneliness, which otherwise does not involve pathological conditions when transient^48^. As shown in the last regression model where the relationship between health conditions and loneliness was substantially reduced after introducing the regressors for depression and feelings of hopelessness, depression-like symptoms induced by prolonged social isolation represent a central factor to the negative effects of loneliness on health.

The observed relationships between prosocial motives and feelings of loneliness seem to support the hypothesis that loneliness facilitates prosocial behaviors. However, such prediction needs still to be validated by future studies. In fact, given the nature of the current study's data (i.e., self-reports) only an association between individual attitudes toward prosocial behaviors and subjective feelings of loneliness could be tested in this work. Individual attitudes have indeed been shown to predict actual behaviors^49,50^. For instance, expectations that a person is lonely induce less sociable behaviors toward the target of such expectations^51^. Nonetheless, attitudes and behaviors do deviate in many occasions and a previous meta-analysis indicate specific conditions under which attitudes and behaviors align^52^. Future longitudinal and experimental studies are hence needed to acquire measurements of social preferences and behaviors (e.g., by using questionnaires such the social value orientation scale and economic games such as the investment game^53,54^) to examine the relationships between loneliness and actual behavior, and to tease apart the different mechanisms and effects of loneliness on behavior and health.

Taken together, these results are the first evidence on the complex psychological dynamics between loneliness, prosocial attitudes and individual expectations about interacting partners. The strong relationship between prosocial preferences and loneliness was able to predict feelings of loneliness of out-of-sample individuals better than relevant biographical variables. Future studies are needed to replicate and extend these findings, for instance, by investigating how the relationship between individual prosocial motives and actual prosocial behaviors is modulated by loneliness. Intriguingly, these results are in line with previous evidence suggesting that gender differences in altruistic behaviors might be related to differences in feelings of loneliness. In particular, consistent with previous evidence (e.g.,^55,56^, but see also:^57^), in the current work, women reported to feel lonelier than men and such difference in feelings of loneliness might explain why women tend to be more altruistic than men^58^. However, it has to be noted that differences between men and women in both reported feelings of loneliness and altruistic behaviors might be due to internalized sex roles expectations. For instance, even though women report higher levels of loneliness than men in an explicit measure of loneliness, men turn out to be lonelier than women in an implicit measure of loneliness, likely because disclosing loneliness is less socially acceptable and more stigmatized for men than women^59–61^. Similarly, women who are less likely to identify themselves with traditionally feminine attributes behave as altruistically as men^62^. These results suggest that social expectations about others are also sensible to the target of those expectations (e.g., the traits and gender of the other person)^63^ and future studies need to control for these differences.

Overall, this work points to an important gap in the literature on loneliness, stresses the central role of loneliness in social motivation and highlights the need for new research avenues to explain how loneliness can, on the one hand, increase an individual’s motivation for social connections but, on the other, also strengthen a propensity to social withdrawal. The cross-validation procedure indicates that social preferences are able to predict subjective feelings of loneliness of out-of-sample individuals, pointing to the importance of these factors to better estimate who might be susceptible to develop loneliness and determine who might fall into a chronic loneliness state. Ultimately, as loneliness is one of the strongest predictors of depression onset, taking into serious consideration the social factors that contribute to the formation and development of loneliness might help predict, and eventually prevent, the onset of more profound depressive states.

## Methods

### Sample

Data were analyzed from the 2017 “Social Networks and Social Resources” survey of the International Social Survey Programme (ISSP; https://www.issp.org/about-issp/). The ISSP is a continuous program of cross-national, annual surveys in 50 countries across the world. All methods were carried out in accordance with relevant guidelines and regulations. The ISSP source questionnaires are developed and pretested by international teams and discussed and approved by the ISSP General Assembly (GA), which is the main deliberative, decision making and representative organ of the ISSP. The GA approves questions based on their scientific merit, sociopolitical relevance and ethical appropriateness. ISSP members, the national field questionnaires and field work, all must comply with the given legal requirements in each country. Before depositing data to the ISSP Archive national ISSP data are anonymized so that individual survey participants cannot be identified. Informed consent was obtained from all subjects. After excluding missing values, data from 15,430 individuals from 15 countries were eligible for analysis.

### Material and statistical analysis

Four hierarchical regression models were fitted by incrementally adding new predictors of subjective feelings of loneliness estimated on the basis of three items. Predictors were divided in four groups: (1) social preferences; (2) quality of social network; (3) health condition and life satisfaction; and (4) mental health.

The three items of loneliness measured: (1) feelings of lacking companionship; (2) feelings of being isolated from others; and (3) being left out. All items are answered on a scale from 1 (never) to 5 (very often).

Items on social preferences included: (1) beliefs about others’ fairness asking participants whether they believe that people try to take advantage of others or be fair on a scale from 1 (take advantage almost all the time) to 4 (try to be fair almost all the time); (2) beliefs about others’ trustworthiness asking participants whether they believe that people can be trusted on a scale from 1 (people can almost always be trusted) to 4 (you can't trust others), which was reversed for analysis; (3) altruistic motives asking participants whether they believe they should take care of themselves before helping others on a scale from 1 (strongly agree) to 5 (strongly disagree); (4) compliance with a reciprocity norm asking participants whether they believe that favors should be repaid on a scale from 1 (strongly agree) to 5 (strongly disagree), which was reversed for analysis; (v) and support of social network on a scale from 1 (strongly agree) to 5 (strongly disagree), which was reversed for analysis.

Items on quality of social network included: (1) frequency of meaningful social contact asking participants how often they go out with friends and acquaintances on a scale from 1 (daily) to 8 (never), which was reversed for analysis; (2) contact seeking asking participants how often they make new friends on a scale from 1 (never) to 5 (very often); (3) and overall frequency of social contact asking participants with how many people they have contact in a weekday on a scale from 1 (0–4 people) to 6 (100 people or more).

Items on health condition and life satisfaction included: (1) general health status on a scale from 1 (excellent) to 5 (poor), which was reversed for analysis; and (2) overall life satisfaction on a scale from 1 (completely satisfied) to 7 (completely dissatisfied), which was reversed for analysis.

Items on mental health included: (1) feelings of depression asking participants how often they feel depressed on a scale from 1 (never) to 5 (very often); (2) and feelings of hopelessness asking participants how often they feel they are unable to overcome difficulties on a scale from 1 (never) to 5 (very often).

Finally, the following measures of biographical variables were included: (1) sex (1 = female; 0 = male); (2) age; (3) education on a scale from 0 (no education) to 6 (upper level tertiary such as Master or Doctor title); (4) urban living on a scale from 1 (big city) to 5 (a farm or home in the country), which was reversed for analysis; (5) employment (1 = employed; 0 = unemployed); (6) and relationship status (1 = single; 0 = in a relationship). All variables were mean-centered and standardized before entering them into the regression models. Biographical variables were included in all models. Hierarchical regression models with country as group variable were run using *fitglme* in MATLAB R2018a (http://www.mathworks.com).

To test the predictive performance of the variables of interest (i.e., social preferences), a cross-validation procedure with bootstrapping was carried out. In particular, random samples corresponding to 20%, 50% and 80% of the original sample size were drawn from the original dataset and used as training data. The model with only regressors for social preferences (social model) was fitted to these training data and its predictive performance was tested on the left-out data (test data). The model was fitted to a total of 10,000 random samples for each of the three different sample sizes and tested to the corresponding left-out data (test data), yielding a total of 30,000 predictions. For each of these predictions, the prediction error (i.e., standardized mean square error) was computed resulting in a distribution of the model’s predictive performance. This distribution was compared to two distributions of predictive performance of two competing models. One was a null model (the permuted model 1) fitted to permutated labels (i.e., loneliness scores) representing a prediction chance level and the other was a model with only biographical regressors fitted to the true labels. For each of these models, the same cross-validation procedure with bootstrapping was applied to compute the distributions of their predictive performance. Finally, models’ predictions were further compared with the true labels by computing Pearson correlations.
